# Implementation of accelerated research: strategies for implementation as applied in a phase 1 Ad26.ZEBOV, MVA-BN-Filo two-dose Ebola vaccine clinical trial in Uganda

**DOI:** 10.1080/16549716.2020.1829829

**Published:** 2020-10-19

**Authors:** Jonathan Kitonsa, Abu-Baker Ggayi, Zacchaeus Anywaine, Eva Kisaakye, Laura Nsangi, Vincent Basajja, Mary Nyantaro, Deborah Watson-Jones, Georgi Shukarev, Ine Ilsbroux, Cynthia Robinson, Pontiano Kaleebu

**Affiliations:** a Medical Research Council, Uganda Virus Research Institute and London School of Hygiene and Tropical Medicine Uganda Research Unit, Entebbe, Uganda; b London School of Hygiene and Tropical Medicine, London, UK; c Janssen Vaccines and Prevention B.V., Clinical Development, Leiden, The Netherlands; d Janssen Research & Development, Portfolio Delivery Operations, Global Development, Beerse, Belgium

**Keywords:** Ebola virus disease, expedited approval, accelerated conduct, vaccine research

## Abstract

**Background:**

The 2013–2016 Ebola epidemic in West Africa is the worst ever caused by *Ebolaviruses* with over 28,000 human cases and 11,325 deaths. The World Health Organisation (WHO) declared the epidemic a public health crisis that required accelerated development of novel interventions including vaccines. The Medical Research Council/Uganda Virus Research Institute and London School of Hygiene and Tropical Medicine Uganda Research Unit (MRC/UVRI & LSHTM Uganda Research Unit) was among the African research sites that implemented the VAC52150EBL1004 Ebola vaccine trial.

**Objective:**

We report on the strategies utilised by the Unit and sponsor in ensuring expedited clinical trial approval and accelerated conduct.

**Methods:**

Janssen Vaccines and Prevention B.V. conducted a phase 1 trial to evaluate the safety, tolerability, and immunogenicity of heterologous two-dose vaccination regimens using Ad26.ZEBOV and MVA-BN-Filo, in healthy adults in Africa. Accelerated implementation strategies are hereby presented.

**Results:**

Strategies included: holding the African Vaccine Regulatory Forum (AVAREF) joint review meeting; expedited review by institutional ethics and country-specific regulatory bodies; competitive recruitment between sites; electronic data capture (EDC); frequent study monitoring schedule; involvement of a community advisory board (CAB); and utilization of a ‘phased’ study information-sharing approach in community engagement and participant recruitment. These strategies enabled the site to acquire approvals within 2 months and enrol 47 participants within a spurn of five. The same milestone is usually acquired in at least 1 year without accelerated implementation.

**Conclusion:**

The use of well-thought strategies by sponsors and research sites can enable the implementation of accelerated research. We recommend the use of similar strategies in other settings.

## Background

The Ebola epidemic of 2013–2016 was the largest the world has ever experienced. Over 28,600 human cases and 11,325 deaths were reported across Guinea, Liberia, and Sierra Leone, during this period [1]. The epidemic was declared a public health crisis in August 2014 by the WHO [2] and necessitated accelerated development of novel interventions including vaccines [3]. WHO called for fast-track development of Ebola vaccines as part of the Ebola Response Roadmap [3] and further stressed in October 2014 that this was a public health priority [4,5]. A WHO panel considered the West African Ebola epidemic extraordinary for several reasons including, but not limited to, its magnitude, contagiousness and high lethality of the disease, and the additional burdens on fragile health systems. All these factors created an opportunity to investigate the disease during an epidemic. The occurrence of these factors led to the consensus that clinical trials had to be conducted urgently [6]. Other international agencies such as the Wellcome Trust and Centre for Infectious Disease Research and Policy brought together experts from all over the world to support the initiative [7]. This collaboration established a team of experts from different backgrounds to rapidly assess challenges and opportunities related to vaccine development, identify potentially overlooked aspects of the vaccine development process, and urgently synthesise information for distribution in the public health domain. Accelerated vaccine development was also partly possible because many vaccine candidates had already been evaluated in pre-clinical studies and many had shown good immunogenicity profiles [5,8].

Subsequently, several clinical trials were conducted during and after the epidemic, many of which were multi-centre international projects using different vaccine candidates [9,10]. One of these was the VAC52150EBL1004 Ebola vaccine clinical trial sponsored by Jansen Vaccines and Prevention B.V. The trial was implemented at the MRC/UVRI & LSHTM Uganda Research Unit site in Masaka, and the Mwanza Intervention Trials Unit in Tanzania [11].

The accelerated implementation of such a research project in Africa was a new approach. It required implementation by well-established research institutions and the use of forward-thinking research processes to accelerate regulatory submissions, to foster robust community engagement, and to initiate and maintain participant recruitment, management, and follow up. This all happened in the context of a background characterized by mixed feelings including fear and scepticism about the use of Ebola vaccines, which had never been extensively used in humans [12–14]. Despite this situation, the sponsor together with the MRC/UVRI & LSHTM Uganda Research Unit was able to conduct the trial within the stipulated timelines. We believe that this accomplishment offers a learning opportunity for the implementation of similar projects in the future in this and other settings, especially where an accelerated approach may be required.

## Methods

### Trial design

VAC52150EBL1004 was a randomized, observer-blind placebo-controlled, trial evaluating the safety, tolerability, and immunogenicity of a two-dose vaccination regimen using Ad26.ZEBOV and MVA-BN-Filo administered in different sequences and schedules in healthy adults (based on clinical and laboratory parameters) [11]. The study enrolled participants aged ≥18 to ≤50 years, who were able to read and provide consent. Participants were excluded if HIV positive, pregnant or breastfeeding or planning to become pregnant within the study execution period. Other details on study eligibility, randomisation, blinding, vaccination, and follow up are published elsewhere [11,15].

Briefly, the study consisted of a screening period of up to 28 days, a vaccination period in which participants were vaccinated on day one, followed by a second vaccination 4 or 8 weeks later and a post-dose 2 follow-up for 21 days, and thereafter follow up for up to 1 year after randomisation.

### Study implementation at the Ugandan site

Regulatory approvals were attained within a spurn of approximately 2 months. The site study initiation visit was conducted on 6 March 2015, participant screening started on 20 April 2015, and the first participant was dosed on 4 May 2015. The site administered the last participant with the second dose on 10 November 2015. Enrolment took place across a spurn of 5 months (from the time screening started to the time the last participant was enrolled). The last participant completed follow up on 16 September 2016 and the site study close-out meeting held on 30 November 2017.

While it took a total of approximately 7 months to attain approval and complete enrolment, it normally takes a minimum of 1 year at the unit for a similar number of participants without accelerated implementation.

The mean age (standard deviation) of the 47 participants enrolled was 25.4 years (5.3), with 34 (77.3%) of these males and 13 (27.7%) females. All 47 participants enrolled completed follow up.

### Review of strategies for successful accelerated implementation

The study sponsor and the MRC/UVRI & LSHTM Uganda Research Unit utilised several strategies to enable accelerated implementation. The sponsor-initiated strategies included: 1) holding the AVAREF joint ethics, regulatory authority, investigator and sponsor review meeting, 2) advocating for expedited review by Institutional ethics and country-specific regulatory bodies, 3) competitive recruitment between sites, 4) electronic data capture, and 5) frequent study monitoring. The MRC/UVRI and LSHTM Uganda Research Unit specific strategies included: 1) involvement of a community advisory board (CAB) and 2) ‘phased’ study information-sharing sessions in community engagement and participant recruitment. These strategies are hereby discussed including details on how each was implemented, rationale for use, lessons learnt, and challenges faced while using the strategy. Appendix 1 is a flow diagram showing the number of participants at different stages of the study and the interplay of strategies, while Appendix 2 is a table summarising the strategies, rationale for using them, the impact, lessons learnt, and challenges met while using them.

### The AVAREF joint ethics, regulatory authorities, investigator and sponsor review meeting

This meeting took place on 3 and 4 February 2015 in Arusha, Tanzania. The objective was to allow joint preliminary review of the Janssen Ebola Zaire vaccine clinical trial applications by ethics and national regulatory authorities of the countries of Ghana, Kenya, Tanzania, and Uganda. The meeting involved presentation of the study protocols by the sponsor and response to questions raised by regulators and other stakeholders including the FDA. Implementing sites also made presentations to demonstrate their readiness to conduct the studies. Finally, regulatory authorities offered their recommendations and agreed upon a commitment to issue approval/non-approval within a certain timeframe, which informed amendments to the study protocols and subsequent independent ethics and regulatory review.

#### Rationale for the strategy

Previous reviews written about the regulatory processes in Africa have shown that the region has several of shortcomings in the conduct of these. Specifically, issues include prolonged timelines for reviews, disparities in procedures for reviews and authorizations, and duplication of roles within different authorities [16,17]. The AVAREF network was formed as an informal capacity-building platform aimed at improving the regulatory oversight of interventional clinical trials being conducted in Africa [18]. Over the years, this platform has demonstrated its value in strengthening regulatory and ethics reviews, promoting harmonized standards and approaches, and accelerating the development of vaccines of high public health value. In the meeting held prior to the Ebola vaccine trials, external support was provided by the USA Food and Drug Administration, European Medicines Agency, and Health Canada. This support was necessary because the products that were about to be tested were new and complex, yet it was extremely necessary to have the studies conducted as soon as possible to offer solutions for the ongoing epidemic. The AVAREF process was also important in understanding the local and regional concerns, addressing them, and educating the western partners on what was critical to conducting studies in the region.

The joint review of the applications would make it easier for the regulatory authorities and other stakeholders to offer preliminary feedback regarding the protocols. The process of responding to queries by the sponsor would be made quicker while recommendations would also be aggregated and acted upon by the sponsor at the time of the meeting. In a nutshell, the AVAREF meeting was not set up to coerce or undermine local regulatory authorities, but to support and eventually give the best opinion about protocol applications.

#### Lessons learnt

Joint sponsor, ethics, and regulatory reviews of study protocols in multi-centre studies as was done in this case is feasible and makes the subsequent process of approval faster upon receipt of the revised protocol and related regulatory documents.

#### Challenges

The different stakeholders attending would have to put aside other responsibilities to come and hold face-to-face discussions during the AVAREF meeting. This was, however, extremely important given the gravity of the disease at hand. Stakeholders would have to subjugate their national viewpoint to accommodate the larger, regional input. This did not however happen coercively, but after discussion. For example, as noted above, while each country and authority had its standards and requirements, it was realised from this meeting that some of these were being duplicated within the same country. This meeting also challenged the viewpoint that reviews always had to be done in sequence even in times of epidemics. The possibility of joint reviews at such times was underscored. The other viewpoint held by some local authorities was that the sponsor and the authorities could not discuss applications together. It was however realised that this is very possible and does not necessarily bias the decision of the authorities.

### Expedited review by institutional ethics and country-specific regulatory bodies

The initial submission to Uganda Virus Research Institute Research and Ethics Committee (UVRI-REC) took place on 13 January 2015 with request for expedited review. Initial feedback from UVRI-REC was received on 19 January 2015 which is within the council’s stipulated 60-day timeline, i.e. the period within which the council indicates they must normally give feedback according to their guidelines [19]. This happened before the AVAREF review meeting that took place on the 3 and 4 February 2015. A second submission was made on 13 February 2015 after a protocol amendment (dated 9 February 2015) following the AVAREF review meeting and final approval was received on 5 March 2015. Application to National Drug Authority (NDA) was made on 5 March 2015 with request for expedited review, and approval was received on 18 March 2015, which is within the 30 day working days timeline stipulated in the authority’s guidelines [20]. Application to Uganda National Council of Science and Technology with request for expedited review was made on 6 March 2015 and approval received on 18 March 2015, which is within the 10 day working days timeline stipulated in the council’s guidelines [20].

#### Rationale for the strategy

The need to conduct this research in an expedited manner required that approvals be attained as soon as possible. This was emphasised in the application letters. The review of the protocol in the AVAREF meeting by several stringent health authorities would give confidence to the Ugandan authorities in the subsequent review process.

#### Lessons learnt

African regulatory authorities have the capacity to review research protocols in an expedited manner and support the implementation of important research including research during epidemics. Interaction between the sponsor, investigators, regulatory authorities, and other stakeholders before formal accelerated protocol submissions creates mutual trust and enlightens a common goal that needs to be achieved by all.

#### Challenges and mitigation

Expedited review carried extra costs for the sites rendering the process more expensive than usual. Making these submissions conferred more responsibility to the site, requiring overtime hours. The impact of extra cost and overtime was mitigated by the team’s experience in research implementation together with strong commitment to making the project a success. Expedited review necessitated streamlined communication between the authorities, implementing site and sponsor, requiring cooperation and personnel availability from all involved to enable the process to flow smoothly. Communication between parties was almost daily and advance notification of any regulatory submissions was provided. Also, the Uganda NDA was particularly eager to hold face-to-face meetings with the site or sponsor which greatly advanced understanding and resolution of issues.

### Electronic data capture

The study was designed to use EDC, particularly utilizing Medidata Rave® [21]. This is a web-based clinical data management system used to capture, manage, and report clinical research data. The system provided various advantages including timely data entry and review, enabling quick decision-making, maximum control, accessibility for study team members, flexibility, easy data extractability, and availability of ad hoc reporting tools. It offered a full query and source document verification suite, which enabled the sponsor to conduct remote monitoring, supplementing onsite monitoring. This reduced the number of errors, queries, and protocol deviations/violations. Medidata Rave ® integrates a dynamic laboratory range management system with laboratory range alerts and local references, enabling instant feedback whenever out of range results were entered. Automatic data cleaning/verification, review, locking and unlocking were also made possible.

#### Rationale for the strategy

The decision to use this system was based on the functions discussed above. In addition to these, the system would enable the sponsor to collaborate data collection, review, feedback, cleaning, and utilization including interim analyses across multiple sites.

#### Lessons learnt

This was the first time this study team used EDC. The site previously used paper-based source documents which would be entered into a database sometimes days after collection. However, the site staff adapted easily with training and orientation. This highlights the importance of maintaining a well-qualified, trained, experienced, and highly motivated study team. EDC is an efficient mode of data management and may be considered for use in other multi-centre trials.

#### Challenges

The challenges were minimal and minor. The commonest was staff forgetting their login credentials because of frequent (three-monthly) expiration of passwords. The effects of this were mitigated by availability of multiple staff with similar roles and availability of various Medidata customer help options including fax, email, and telephone. In beginning, no standard operating procedures were available on the use of EDC. EDC was set up based on guidelines offered by the sponsor and detailed in an EDC set up and user guide. However, SOPs subsequently had to be put in place that would apply to this and other studies that would later be conducted at the unit.

### Competitive recruitment between sites

This trial was designed in such a way that two different sites in different countries implemented the same protocol. Masaka site in Uganda was paired with Mwanza Intervention Trials Unit in Tanzania, with both sites required to recruit a total of 72 participants. While this strategy has been used elsewhere, it had not been used often in previous trials the unit participated in. The more common practice was for the unit to have a defined ration of participants to enrol.

#### Rationale for the strategy

This was intended to achieve rapid recruitment amidst anticipated ethical and operational challenges that could occur at either site. The other reason for this strategy was to generate safety and immunogenicity data from demographically divergent populations. Data would inform where to best conduct subsequent phase 2 and 3 studies.

#### Lessons learnt

Competitive recruitment of volunteers between different sites and regions can help mitigate hindrances related with conducting accelerated research including challenges with ethical and regulatory clearance and institution-specific operational issues. This is because as one site continues to work its way through these requirements as well as other challenges that may hinder recruitment, another site in a different country operating under different circumstances can manage to proceed.

#### Challenges

Challenges faced at one site can cause un-anticipated need to make re-adjustments at the other site such as the requirement to enrol more participants than previously planned. The site in Tanzania had issues with recruiting the planned number of eligible participants, requiring that the site in Uganda recruit eleven more participants. This inevitably came with the need to make budgetary and logistical adjustments.

### Frequent monitoring schedule

The study monitors were required to conduct frequent visits according to the study protocol. Initially, the monitors used to spend 2 weeks at the site monthly. They were also required to be on site for the first vaccination to ensure that procedures went as required per protocol and offer guidance if required. There was a standby ‘flying squad’ of monitors from Europe that would come to the site whenever the workload would increase. In addition to these frequent onsite-monitoring visits, investigational product handling aspects were also frequently reviewed by a separate team of monitors who would interact with the site pharmacy team and not the clinical team.

#### Rationale for this strategy

This accelerated Ebola vaccine development program was intended to generate safety and immunogenicity data on the vaccine candidate within a short period before proceeding to Phase II and III trials. This monitoring strategy was intended to ensure compliance with the protocol, good clinical practice, and applicable standard operating procedures. Queries would also be generated and handled with no delays, while the presence of monitors on-site would also offer another quick mechanism of communication between the sponsor and the study team.

#### Lessons learnt

This strategy partly prevented the site from making multiple protocol deviations. We recommend that it is adopted even in ordinary non-accelerated clinical trials (even though the schedule may be less frequent) to avoid resource wastage, recruitment of ineligible participants and other issues that come along with protocol deviations.

#### Challenges

The study staff had to apportion time appropriately between the strenuous recruitment activities and attending to the monitoring queries. More staff were recruited, which in turn increased the cost of running this study.

### Involvement of the community advisory board

The MRC/UVRI & LSHTM Uganda Research Unit routinely utilises support from the CAB before and during the conduct of its research studies. This board is comprised of 20 members including political, administrative, religious, and cultural leaders, medical professionals, local media, community-based organisations, people living with HIV/AIDS, study participants, as well as representatives from the general community.

The CAB was convened for various meetings during which different aspects of Ebola virus disease were discussed, including the status of the Western Africa epidemic and the need for a vaccine to curb the epidemic. Information about the VAC52150EBL1004 protocol was shared and eventually, the study team received feedback from the CAB with members advising on the practicality of implementing the study in this setting. The CAB overwhelmingly agreed that this study was implementable in this setting and that results gained would be of utmost value to everyone especially in Uganda where several Ebola outbreaks had occurred. Particularly important, the CAB advised that given the nature of the study with stringent eligibility requirements such as ability to read and write, it would be wise to engage the wider community, but particularly giving extra focus to educated communities including university students, health workers, and teachers.

#### Rationale of the strategy

Previous controversies in the conduct of biomedical research worldwide led to calls from bioethicists to mandate community involvement in decision-making about the ethical conduct of health research [22,23]. Participation of community representatives at different levels of research implementation is required to ensure that communities are not exploited [24]. Other benefits of having community representatives included: help discover what the specific local concerns were, squash rumours, enlist support of community leaders, express the community’s desire to participate in the research, elicit information from researchers that the community may need to know about the study, participation in dissemination of research findings, among others [25]. CABs have been used to provide these functions at the MRC/UVRI & LSHTM Uganda Research Unit in multiple research projects and this relationship over the years has contributed to successful implementation of research.

#### Lessons learnt

While the practice of using CABs has been prevalent over the years at the unit, its usefulness in this study needs to be commended. The idea of focusing on educated communities such as university students, health workers, and teachers was forwarded by the CAB. The functions of a CAB remain paramount even in the implementation of accelerated research and especially research on diseases that spread an aura of fear into communities.

#### Challenges

The limitation on time implied that meetings with the CAB had to be organised urgently. However, flexibility from both the researchers and CAB made these meetings successful. Study personnel also had to communicate study information in a manner that CAB members of diverse backgrounds could comprehend. With the help of various communication aids and extensive experience on the side of both researchers and the CAB, key messages were communicated by the study team and clearly understood by the CAB members.

### The phased study information-sharing approach in community engagement and participant recruitment

The site used a phased study information-sharing approach culminating in screening, with all sessions including screening occurring between April and August 2015. We have referred to this approach as ‘phased’ because information was shared in different sessions that built onto one another regarding information shared. We hereby describe the issues discussed in each session.

**Session 1**: These sessions were held at centres in the community including teaching institutions, health facilities – particularly targeting health workers; and community halls – targeting other community members. These sessions focussed on haemorrhagic fever diseases with emphasis on Ebola virus disease (EVD). Issues discussed included what EVD is, the case fatality rates, its history with emphasis on previous epidemics in Uganda, modes of transmission, signs and symptoms, and how it can be prevented. It was stressed that new interventions were required, including the use of vaccines, to prevent and treat haemorrhagic diseases. At the end of the presentations, attendees had the opportunity to ask questions. Those interested in learning more about potential treatments and vaccines were requested to register for another session. Overall, 270 potential volunteers attended these sessions, 177 of these (65.5%) being male, with each session registering an average of 50 potential volunteers.

**Session 2**: These sessions were held at the MRC/UVRI & LSHTM Uganda Research Unit site in Masaka. Of the 270 potential volunteers who attended the first session, 233 (86.3%) returned for the second session, 149 (63.9%) being male. Issues discussed in this session included potential treatments and vaccines. Emphasis was on vaccines, including a review of the different vaccine types, how vaccines are manufactured, and progression of trials through pre-clinical to human stages. The VAC52150EBL1004 trial was briefly discussed, including how this had so far progressed through the preclinical development stages, and preliminary results from a similar study implemented under a different protocol but using the same vaccine candidates in the UK [26]. Volunteers could ask questions at the end of the presentations, after which registration was open for those interested in returning for a final session.

**Session 3**: This session also took place at the study site. Out of the 233 potential volunteers who attended the second session, 180 returned for this session, 114 (63%) being male. Each member was individually taken through the study participant information sheets (PIS) and a consent form by designated study staff. This was followed by answering a test of understanding comprising 10 objective questions in the same language as the PIS and consent form (English or Luganda). Volunteers that scored at least 9/10 on at most two attempts were given the opportunity to sign the consent form if they wanted to proceed with the study. Out of 180 volunteers, 176 (98%) passed the test of understanding and all of these signed the consent form.

Participants that offered consent proceeded to a screening phase. Of the 176 that entered screening, 47 participants met the eligibility criteria and were enrolled into the study (Screening to enrolment ratio of 4:1). The site completed this process in approximately 5 months.

#### Rationale of the strategy

Given the sensitivity around the idea of vaccination against an extremely deadly disease using genetically modified virus components, potential volunteers needed time and multiple interactions to correctly process the complicated information. We considered it necessary to give them time to think critically about the information and ask questions before making a final decision to participate in the study. Due to the complexity of the information, we considered that breaking it down would make it easier to communicate and understand. We also considered that those that would attend the sessions but not eventually participate would generally be educated about EVD; therefore, the sessions also served as Ebola awareness campaigns.

#### Lessons learned

This strategy made it easier to communicate sensitive information and eventually enabled the site to complete recruitment in only 5 months. The idea of initially meeting potential participants in the community made it easier for many to attend without necessarily having them feel like they were mandated to participate in the trial.

#### Challenges

This strategy was expensive due to the requirement to reimburse all attendees. In addition, the site had to get ethical approval for all the materials that were used during these sessions.

## Conclusion

The use of well-thought methods designed *a priori* by the sponsor and site enabled the team at MRC/UVRI & LSHTM Uganda Research Unit to successfully implement this phase one research project in an accelerated manner and within the stipulated timelines. Emerging and re-emerging disease threats that require the development of more potent drugs and vaccines are increasing. Fortunately, some outbreaks take a short period to abate or stop completely, however that would mean missing the opportunity to evaluate new interventions. Research teams need to embrace efficient strategies to implement research within the small window of disease outbreaks.

## Recommendations

We recommend that other research teams planning to participate in the implementation of accelerated research especially in resource-limited settings consider utilising similar strategies. Accelerated research requires that regulatory authorities and research teams establish strong working relationships so that approvals are attained promptly. Community engagement remains key, and methodologies to communicate sensitive research issues have to be considered in order to deliver the messages appropriately. The implementation of this kind of research requires that research facilities adapt to the use of electronic data collection tools, while continuous oversight of trials through frequent monitoring makes data cleaning a continuous and timely process and reduces on occurrence of protocol deviations.

## Paper context

The 2013-2016 Ebola epidemic in West Africa required accelerated development of interventions including vaccines. MRC/UVRI & LSHTM Uganda Research Unit implemented the VAC52150EBL1004 trial in an accelerated manner, using various strategies such as holding the AVAREF review meeting; expedited review by institutional regulatory authorities, and involvement of a community advisory board. This enabled enrolment to be completed within 5 months. The use of similar strategies in other settings may enable accelerated research in epidemics.

## Appendix 2. Table summarising strategies

**Implementation of accelerated research: strategies for implementation as applied in a phase 1 Ad26.ZEBOV, MVA-BN-Filo two-dose Ebola vaccine clinical trial in Uganda**

**Table:**

Strategy	Rationale for strategy	Impact of using strategy	Lessons learnt	Challenges
Holding the African Vaccine Regulatory Forum (AVAREF) joint review meeting.	-Enlist the support of external agencies such as the Food and Drug Administration in conducting review of applications. -Harmonise the roles of regulatory authorities in the conduct of reviews thereby avoiding duplication of roles. -Enable regulatory authorities to communicate their opinions about this type of research. -Inform western partners about local/regional requirements in the conduct of research. -Enable joint review of protocols and enable quicker response to queries. -Communicate recommendations to the sponsor.	-Made subsequent independent reviews easier and quicker. -Clarified local/regional needs to the sponsor.	-Underscored the importance of joint protocol review especially in the setting of a wide spread epidemic.	-Time commitment from all stakeholders. -Requirement to harmonise requirements.
(2)Expedited review by institutional ethics and country-specific regulatory bodies.	Need to have research conducted very first to respond to an emergency at hand.	Enabled research to begin quickly.	-Capacity is available among local regulatory authorities to do expedited review. -Interactions between the sponsor and authorities creates mutual trust.	-Higher fees had to be paid by the site to the authorities for expedited review. -More commitment than usual required of sites to make submissions quickly.
(3)Electronic data capture (EDC).	-Need for timely data entry and review. -Need for quick data guided decision making. -Need to easily generate and respond to queries.	-Timely data entry and review enabled. -Enabled real time tracking of safety data and timely intervention. -Enabled remote monitoring to supplement onsite monitoring.	-Underscored the use of similar platforms in data management. -Advantages realised including remote monitoring make the use of this system extremely necessary, especially in times of epidemics/pandemics where movement to and research sites may not be very easy.	-Staff occasionally forgot log in credentials. -Data locks would require that individuals with unlocking rights are contacted whenever changes had to be made to data. A bit of time was wasted during such intervals. -Standard operating procedures for use of EDC were initially not available. These had to subsequently be designed.
(4)Competitive recruitment between sites.	-More generalizable data taking advantage of demographic variations would be generated. -Challenges in recruitment at one site would be counteracted.	When the Mwanza site got challenges, our Ugandan site was in position to recruit an extra number of participants.	Competitive recruitment can help mitigate challenges encountered at one site by having the other site make re adjustments.	Need to quickly plan and make budgetary adjustments.
(5)Frequent study monitoring schedule.	-Ensure compliance to the protocol, GCP, and applicable standard operating procedures. -Timely generation and response to queries.	Enabled generation of timely clean data for decision making.	-This strategy can help reduce on the occurrence of protocol deviations/violations which could have serious consequences for participants and the study in general.	-Increased trial running costs. -More commitment required from staff attending to issues/sorting queries.
(6)Involvement of a community advisory board (CAB).	-Prevent exploitation of participating communities. -Act as a bridge linking the research site and the community. -Make an input in the design of the study especially as regards the recruitment strategy.	-The CAB gave invaluable inputs that informed recruitment. -Community concerns were communicated by the CAB. -Feedback to the communities was communicated through the CAB.	The CAB is an incredible resourceful group whose inputs make the research process easier.	-Money had to be spent in organising multiple sessions with the CAB.
(7)Utilization of a ‘phased’ study information-sharing approach in community engagement and participant recruitment.	-Need to break down the complicated scientific messages into ‘smaller chunks’ that potential participants would understand. -It would be necessary for potential participants to be given time to deliberate on information passed onto them. -Potential participants needed to be given adequate time to make an informed decision to participate in the study.	-It became easier to communicate the research messages after breaking them into ‘smaller chunks’. -Those that eventually got enrolled understood the study very well as demonstrated on the test of understanding. -General messages related to Ebola virus disease were given to community members.	-This approach is a very good approach for a study that involves a lot of detail. -Starting information sharing in the community ensures that only interested potential participants come to the site.	-Organising multiple sessions increases financial expenditure. -Ethical approval was required for all communication aids. Finding ideal settings in the community where presentations could be made was sometimes challenging.
